# AI-Assisted Detection and Localization of Spinal Metastatic Lesions

**DOI:** 10.3390/diagnostics14212458

**Published:** 2024-11-03

**Authors:** Edgars Edelmers, Artūrs Ņikuļins, Klinta Luīze Sprūdža, Patrīcija Stapulone, Niks Saimons Pūce, Elizabete Skrebele, Everita Elīna Siņicina, Viktorija Cīrule, Ance Kazuša, Katrina Boločko

**Affiliations:** 1 Faculty of Medicine, Rīga Stradiņš University, LV-1010 Riga, Latvia; 040214@rsu.edu.lv (K.L.S.); 040230@rsu.edu.lv (P.S.); 032155@rsu.edu.lv (A.K.); 2 Faculty of Computer Science, Information Technology and Energy, Riga Technical University, LV-1048 Riga, Latvia; arturs.nikulins@edu.rtu.lv (A.Ņ.); niks-saimons.puce@edu.rtu.lv (N.S.P.); 3 Faculty of Civil and Mechanical Engineering, Riga Technical University, LV-1048 Riga, Latvia; elizabete.skrebele@edu.rtu.lv; 4 Faculty of Biology, University of Latvia, LV-1004 Riga, Latvia; everita.elina@biomed.lu.lv; 5 Department of Radiology, Faculty of Medicine, Rīga Stradiņš University, LV-1010 Riga, Latvia; vikdem@rsu.lv; 6 Department of Computer Graphics and Computer Vision, Riga Technical University, LV-1048 Riga, Latvia; katrina.bolocko@rtu.lv

**Keywords:** artificial intelligence, spinal metastases, vertebrae segmentation, computer tomography, medical imaging, instance segmentation, radiomics

## Abstract

Objectives: The integration of machine learning and radiomics in medical imaging has significantly advanced diagnostic and prognostic capabilities in healthcare. This study focuses on developing and validating an artificial intelligence (AI) model using U-Net architectures for the accurate detection and segmentation of spinal metastases from computed tomography (CT) images, addressing both osteolytic and osteoblastic lesions. Methods: Our methodology employs multiple variations of the U-Net architecture and utilizes two distinct datasets: one consisting of 115 polytrauma patients for vertebra segmentation and another comprising 38 patients with documented spinal metastases for lesion detection. Results: The model demonstrated strong performance in vertebra segmentation, achieving Dice Similarity Coefficient (DSC) values between 0.87 and 0.96. For metastasis segmentation, the model achieved a DSC of 0.71 and an F-beta score of 0.68 for lytic lesions but struggled with sclerotic lesions, obtaining a DSC of 0.61 and an F-beta score of 0.57, reflecting challenges in detecting dense, subtle bone alterations. Despite these limitations, the model successfully identified isolated metastatic lesions beyond the spine, such as in the sternum, indicating potential for broader skeletal metastasis detection. Conclusions: The study concludes that AI-based models can augment radiologists’ capabilities by providing reliable second-opinion tools, though further refinements and diverse training data are needed for optimal performance, particularly for sclerotic lesion segmentation. The annotated CT dataset produced and shared in this research serves as a valuable resource for future advancements.

## 1. Introduction

### 1.1. Machine Learning in Healthcare

Radiomics has emerged as a novel discipline within artificial intelligence, focusing on the extraction of malignancy-associated information from medical images by integrating pathophysiologically significant data into mathematical parameters [1]. Clinical integration of radiomics necessitates several key steps. Initially, a clearly defined target population must be established, utilizing radiomics to achieve improvements over standard-of-care diagnostic examinations. Secondly, technical considerations regarding statistical methodologies are paramount; minimizing variability through image stratification and addressing machine learning aspects such as potential biases and computational parameters—illustrated by techniques like Bayesian updating—are essential. Thirdly, the reproducibility of experimental test performance must be ensured to validate findings [2]. Additionally, adherence to regulations such as the European Union’s General Data Protection Regulation ensures transparency and ethical compliance in clinical applications [1].

Deep residual convolutional neural networks have been employed for the detection of metastatic bone lesions through automatically segmented regions. Deep-learning segmentation techniques can be adapted for use in computed tomography (CT) scans and magnetic resonance imaging (MRI) [3]. CT scans offer a sensitivity of up to 74% and a specificity of 56%, providing comprehensive assessment of the skeletal system and systemic staging, while minimizing patient exposure to radioactivity. MRI, on the other hand, delivers superior resolution for both bone and soft tissue, with proposed sensitivity and specificity rates of 95% and 90%, respectively [1].

Bone metastases can be diagnosed using a variety of imaging modalities, each presenting specific advantages and limitations. Plain radiographs (X-rays) are typically the initial imaging method for patients presenting with bone pain; however, they have limited utility in asymptomatic patients and in evaluating bones with a high cortex-to-marrow ratio, such as the ribs, due to their low sensitivity in detecting subtle or early-stage metastases [4]. In such cases, CT scans are preferred, offering superior resolution for cortical and trabecular bone structures and allowing for adjustments in window width and level. CT provides detailed multiplanar views, enhancing diagnostic sensitivity and specificity. A recent study reported a pooled sensitivity of 72.9% and a specificity of 94.8% for CT in detecting bone metastases, particularly in areas like the ribs [5]. In oncology, CT scans are commonly utilized for staging and follow-up in cancers affecting the thorax and abdomen, covering extensive portions of the axial skeleton. This enables clinicians to assess the spread of metastatic disease and differentiate between metastatic and degenerative changes. CT also assists in evaluating structural abnormalities identified using other modalities such as MRI and scintigraphy [6]. Although other imaging techniques like MRI, positron emission tomography/computed tomography (PET/CT), single-photon emission computed tomography (SPECT), and bone scintigraphy offer higher sensitivities (91% for MRI, 90% for PET/CT, and 86% for scintigraphy), their routine use is limited by higher costs and reduced availability, especially in countries with constrained healthcare resources [7]. MRI is typically reserved for cases requiring detailed soft-tissue contrast or assessment of bone marrow involvement, while PET/CT and scintigraphy are employed in specific scenarios but are less common for general screening, due to their expense and logistical challenges [8].

Selecting the appropriate imaging modality based on clinical needs is crucial for optimizing diagnostic accuracy. CT remains an accessible and reliable choice for detecting bone metastases, especially in complex anatomical regions like the ribs, where other imaging techniques may have limited utility. Artificial intelligence is a rapidly advancing technology demonstrating significant potential across various domains, including medicine. AI is poised to transform numerous aspects of the medical field such as patient care, administrative processes, diagnostics, treatment planning, and scientific research. In radiology, AI is often combined with radiomics to extract quantitative features from medical images, uncovering patterns not visible to the human eye. These radiomic features, when integrated with AI methodologies like machine learning and deep learning, enhance diagnostic accuracy, prognostic predictions, and personalized treatment strategies. AI technologies—including machine learning, natural language processing, and robotics—can be applied independently or synergistically to analyze clinical data, generate reports, assist in diagnosing conditions, and predict treatment outcomes based on patient-specific variables. The integration of radiomics and AI holds the potential to refine medical imaging analysis, offering deeper insights into disease characterization and treatment efficacy [9].

Higher diagnostic accuracy not only improves patient outcomes but also prevents unnecessary tests that consume time and financial resources, pose psychological burdens, and may expose patients to ionizing radiation and toxic contrast media. Research indicates that artificial intelligence shows promise, with a high accuracy in diagnostics across various specializations:

- Radiology: Recognition of tuberculosis in chest X-ray images, differentiation of benign and malignant lung nodules based on CT data, detection of breast cancer lesions in mammography, and classification of other tumors.
- Pathology: Differentiation of melanocytic lesions, classification of gastric cancer types, prediction of gene mutations associated with cancer, and determination of kidney function from biopsy results.
- Ophthalmology: Diagnosis of retinal diseases, glaucoma, keratoconus, and grading of cataracts.
- Cardiology: Improvement in cardiovascular risk prediction and patient outcome prediction accuracy in pulmonary hypertension.
- Gastroenterology: Endoscopic detection of colorectal polyps, gastric and esophageal cancer, Barrett’s esophagus, squamous cell carcinoma, and other lesions.

The growing need for healthcare services and the advancement of artificial intelligence have led to the creation of conversational agents—chatbots and speech recognition screening systems—that can assist with various health-related tasks such as behavioral change, treatment support, health monitoring, training, triage, and screening. Studies have generally shown that these conversational agents are effective and satisfactory [10]. While AI has the potential to automate certain tasks in healthcare specialties involving digital information, such as radiology and pathology, it is not expected to replace healthcare specialists. Instead, AI aims to augment their skills, allowing them to focus more on patient care and tasks requiring uniquely human abilities such as empathy, persuasion, and holistic integration of information. The integration of AI into healthcare presents ethical, legal, and practical challenges that must be carefully addressed. Further research is necessary to fully understand the long-term effects and ensure the safe and effective incorporation of AI-based technologies into healthcare systems [11].

### 1.2. Vertebral Metastases

Vertebral metastases represent the secondary involvement of the vertebral spine by hematogenously disseminated metastatic cells [12]. They constitute the third most common site of metastasis, after the lungs and liver, and are a major cause of morbidity, characterized by severe pain, impaired mobility, and pathological fractures [13]. Remarkably, vertebral metastases are asymptomatic in 90% of cases and are present in 60–70% of patients with systemic cancer. Approximately 80% of primary tumors give rise to bone metastases [13], which are classified as osteolytic, osteoblastic, or mixed, according to their primary mechanism of interference with normal bone remodeling. Osteolytic lesions are characterized by the destruction of normal bone, while osteoblastic (sclerotic) lesions involve the deposition of new bone [14].

Primary tumors with predominantly osteolytic metastases include breast cancer (65–75%), thyroid cancer (65–75%), urothelial cancer (20–25%), renal cell carcinoma (20–25%), melanoma (14–45%), non-Hodgkin lymphoma, and multiple myeloma. Types with predominantly sclerotic metastases include prostate cancer (60–80%), small cell lung cancer (30–40%), and Hodgkin lymphoma [12,13,14]. Mixed-type lesions are present in breast cancer (15–20%), gastrointestinal cancers, and squamous cell carcinomas [14]. The incidence of spinal metastatic disease is increasing, due to improved patient survival and advanced diagnostic techniques [15]. Median survival from the diagnosis of bone metastasis varies among different cancers: 6 months in melanoma; 6–7 months in lung cancer; 6–9 months in bladder cancer; 12 months in renal cell carcinoma; 12–53 months in prostate cancer; 19–25 months in breast cancer; and up to 48 months in thyroid cancer [14].

Unfortunately, no treatment has been proven to increase the life expectancy of patients with spinal metastases. The primary goals of therapy are pain control and functional preservation [16]. Therefore, it is crucial, not only to diagnose spinal metastases, but also to monitor disease progression, evaluate the stability of the vertebral column, and identify patients who may benefit from surgical consultation or intervention. Multiple scoring systems are available for evaluating different aspects of well-being in patients with metastatic spine disease. One such system is the Spinal Instability Neoplastic Score (SINS), which is used to assess spinal instability and acts as a prognostic tool for surgical decision-making [17].

The SINS is based on one clinical factor (pain) and five radiographic parameters: location, bone lesion quality, spinal alignment, vertebral body collapse, and involvement of posterolateral spinal elements. Each component is assigned a score reflecting its contribution to the overall instability of the spinal segment. The six individual scores are summed to yield a cumulative score ranging from 0 to 18, with higher total scores indicating more severe instability [18]. In the evaluation of 131 surgically stabilized spine metastasis patients, the SINS demonstrated near-perfect inter- and intra-observer reliability in determining three clinically relevant categories of stability. Patients with a SINS ≥ 7 who underwent surgical stabilization showed significant improvements in quality of life [15].

The presented research serves as a proof of concept for an upcoming project in which we plan to create a representative cohort group with even age, sex, and oncology stage distribution within the dataset. Our main objective is to locate metastases in patient computed tomography (CT) scans, when present. On a daily basis, small, barely visible occurrences of metastases in CT scans can be easily missed by healthcare professionals. A well-tuned artificial intelligence (AI) system that can indicate regions of possible disease could be crucial for patient outcomes. If there is any deformation of the bone morphology, an AI could detect and record it. Tasks that have been performed manually by professionals could be accomplished faster and with greater precision by AI. After the identification of deformation regions, healthcare professionals could determine whether these are metastases.

The U-Net segmentation architecture was initially developed for medical imaging data analysis. Its architecture provides segmentation masks with the same size as the input, which is ideal for indicating possible metastases. For this project, we utilized a 3D version of the U-Net architecture to work with the three-dimensional nature of CT data, along with the 2D version [19,20]. One study demonstrated that a deep-learning algorithm (DLA) could assist radiologists in detecting possible spinal cancers in CT scans. The system, which used a U-Net-like architecture, achieved a sensitivity of 75% in identifying potentially malignant spinal bone lesions, significantly boosting radiologists’ ability to detect incidental lesions that might otherwise go unnoticed due to scan focus or diagnostic bias. In this context, AI serves as a second reader, significantly increasing detection sensitivity, without leading to excessive false positives [21].

Another important component of AI in spinal metastatic imaging is its involvement in early detection and therapy, which is key for avoiding complications and enhancing patient quality of life. Recent research has explored the use of AI approaches in image processing, diagnosis, decision support, and therapeutic assistance, summarizing the current applications of AI applications in spinal metastasis care. These technologies have shown promising results in boosting work productivity and reducing adverse events, but further study is needed to evaluate clinical performance and enable adoption into routine practice [22]. A similar study introduced a deep learning (DL) algorithm designed for diagnosing lumbar spondylolisthesis using lateral radiographs. This research aimed to improve the accuracy of medical diagnostics by assisting doctors in reducing errors in disease detection and treatment. The study was retrospective, involving multiple institutions, and focused on patients with lumbar spondylolisthesis. The DL models utilized included Faster R-CNN and RetinaNet for spondylolisthesis detection, demonstrating the potential of AI to significantly enhance diagnostic accuracy in spinal conditions [23].

## 2. Materials and Methods

Our research was organized into two distinct stages, utilizing a previously validated methodology for the preprocessing of radiological data to enhance precision in detecting spinal metastases [24]. The first stage centers on the localization of the patient’s spine, a critical step for identifying regions potentially impacted by metastatic disease. In this stage, each vertebra is individually isolated and segmented, starting from the cervical spine and extending through to the lower spine, inclusive of the sacrum and coccyx. This meticulous segmentation allows for a detailed anatomical mapping, which is essential for subsequent identification of metastases.

The second stage targets the identification and classification of metastases by applying segmentation masks to identify lytic and sclerotic metastatic lesions within the spine. Our approach leverages two U-Net-based neural networks: the first network is specifically trained for spine localization and segmentation, ensuring each vertebra is accurately detected. The second network is dedicated to the instance segmentation of metastatic lesions, where it not only identifies the lesions but also categorizes them by type, distinguishing between lytic and sclerotic metastases. This structured, multi-network approach ensures highly accurate localization and identification of spinal metastases, thereby supporting targeted clinical interventions.

For this study, we utilized two distinct datasets. The first dataset, intended for vertebra segmentation, comprises CT scans from 115 patients diagnosed with polytrauma but presenting relatively undamaged spines. These full-body CT scans were acquired at the RAKUS (Rīgas Austrumu klīniskā universitātes slimnīca) hospital. The second dataset, focused on metastasis detection, includes CT scans from 38 patients diagnosed with spinal metastases, with the detailed information outlined in Table 1 [25]. This dual-dataset structure allowed for both a robust segmentation framework and reliable identification of metastatic patterns across diverse patient presentations.

In this study, we leveraged the nnU-Net library’s comprehensive, built-in data augmentation capabilities to enhance the model performance and generalizability across diverse imaging scenarios. These augmentation techniques are an integral part of nnU-Net’s adaptive framework and include a wide array of transformations, such as rotations, scaling, Gaussian noise, Gaussian blur, adjustments in brightness and contrast, low-resolution simulation, gamma correction, and mirroring. By utilizing nnU-Net’s robust and automated augmentation pipeline, our model benefited from consistent, optimized data transformations across all training subsets, significantly increasing the data diversity and minimizing overfitting risks. This built-in augmentation framework allowed for seamless integration of complex augmentation strategies, without additional external code, thus ensuring reproducibility and alignment with nnU-Net’s standardized approach.

The augmentation process in nnU-Net is designed to adaptively balance transformation intensity and type based on dataset characteristics, which aligns well with our dual-stage segmentation approach. In the initial stage, the augmentations enhanced the training of the neural network tasked with isolating individual vertebrae from the cervical to the lower spine, including the sacrum and coccyx. In the subsequent stage, augmentations strengthened the model’s capability to distinguish between and accurately segment lytic and sclerotic metastases, particularly given the subtle differences in appearance between these lesion types.

The model architecture itself is based on an encoder–decoder structure with skip connections, facilitating the integration of high-level, semantic features with detailed spatial information. This architecture, optimized for segmentation, employed instance normalization to enhance the data consistency across batches, leaky ReLU activation to introduce non-linearity, and deep supervision with topology-adapted parameters. These architectural choices were specifically designed to accommodate the complex, heterogeneous nature of spinal metastases. Skip connections enabled the network to retain high-resolution information across encoding and decoding paths, which is crucial in medical imaging, where spatial precision directly impacts diagnostic utility.

Furthermore, the training data were meticulously curated with segmentation masks, manually created by medical professionals at Riga Stradiņš University using 3D Slicer software (v5.6.0). This software, known for its capabilities in medical image informatics, image processing, and three-dimensional visualization, allowed for precise segmentation, essential for training accuracy. [26] Following segmentation, the data were converted from DICOM into a “nearly raw raster data” format to optimize input/output operations during model training, allowing for efficient handling of the large image volumes typical in medical imaging.

The nnU-Net framework for hyperparameter selection in biomedical image segmentation utilizes a systematic approach to mitigate the complexities associated with manual configuration, as described in the article by Isensee et al. [27]. The automated parameterization in nnU-Net combines fixed, rule-based, and empirical parameters to achieve optimal adaptability across diverse datasets with minimal human intervention.

Fixed Parameters:

Architecture Template: nnU-Net follows a U-Net-like architecture, based on the hypothesis that a well-configured U-Net remains challenging to surpass in performance. This template employs a plain encoder–decoder structure, with two convolutional blocks per resolution level and instance normalization instead of batch normalization to handle smaller batch sizes (usually 2 for 3D models due to GPU constraints). Patch sizes are large enough to capture contextual information, optimized through an iterative reduction process for efficient GPU utilization.

Training Configuration: Training typically ran for 1000 epochs across 250 minibatches per epoch, employing a learning rate that followed a polynomial decay (initial value 0.01) and was reduced throughout training. The optimization was performed using stochastic gradient descent with Nesterov momentum (μ = 0.99).

Loss Function: nnU-Net utilizes a combined Dice and cross-entropy loss to balance between foreground–background class segmentation accuracy and boundary precision, making it well-suited for various biomedical segmentation tasks.

Rule-Based Parameters:

Dataset Fingerprinting and Target Spacing: For each new dataset, nnU-Net analyzes specific characteristics like voxel spacing and median image shape. For anisotropic datasets (e.g., those with a spacing ratio greater than 3 between axes), nnU-Net employs anisotropic resampling strategies, using the tenth percentile of the lowest resolution axis to maintain structural integrity in resampled images (s41592-020-01008-z).

Patch Size and Network Topology: The initial patch size is determined by the median image shape, and iteratively adjusted to meet GPU memory constraints. Network topology, including the number and configuration of downsampling layers, is adapted according to the target spacing and voxel size, ensuring that the effective receptive field size matches the patch size and contextual requirements (s41592-020-01008-z).

Normalization and Augmentation: nnU-Net applies different normalization techniques based on the modality, with z-score normalization for most cases, but using specific percentile clipping and z-scoring for CT images to retain tissue properties. Data augmentation encompasses a variety of transformations, such as rotations, scaling, noise addition, and mirroring, enhancing the model’s generalization ability across various tasks (s41592-020-01008-z).

Empirical Parameterization:

Post-Processing Configuration: Post-processing decisions, such as the inclusion of largest-component suppression, are empirically tested, to assess whether they improve cross-validation performance. This step ensures that nnU-Net achieves optimal accuracy by removing false-positive predictions in multi-class segmentation tasks.

Ensemble Selection: nnU-Net evaluates the performance of different configurations, including 2D, full-resolution 3D U-Net, and a cascaded 3D U-Net, across cross-validation folds. It then selects the best-performing model or an ensemble of configurations to enhance the predictive robustness.

Following data preparation, four U-Net architecture subtypes were trained: 2D images in the form of single slices from CT scans; 3D low-resolution with downsampled input image data; 3D full-resolution utilizing the original resolution of the CT scans; and 3D cascade full-resolution, which used downsampled images to understand the overall structure at a large scale, before learning details from the full-resolution image data, as stated in the utilized library [27].

## 3. Results

In the first stage, the spine was segmented to support the subsequent analysis of metastatic lesions. The U-Net architecture, trained with five cross-validations, employed a composite loss function that combined Dice loss and cross-entropy loss for optimal segmentation performance. Training was conducted on patches extracted from the original images, and the Dice metric was calculated over these patches to evaluate the segmentation accuracy. During inference, a sliding window approach was utilized, introducing patches that may have differed from those encountered in training, which could have contributed to minor fluctuations in the Dice score.

**Figure 1:**
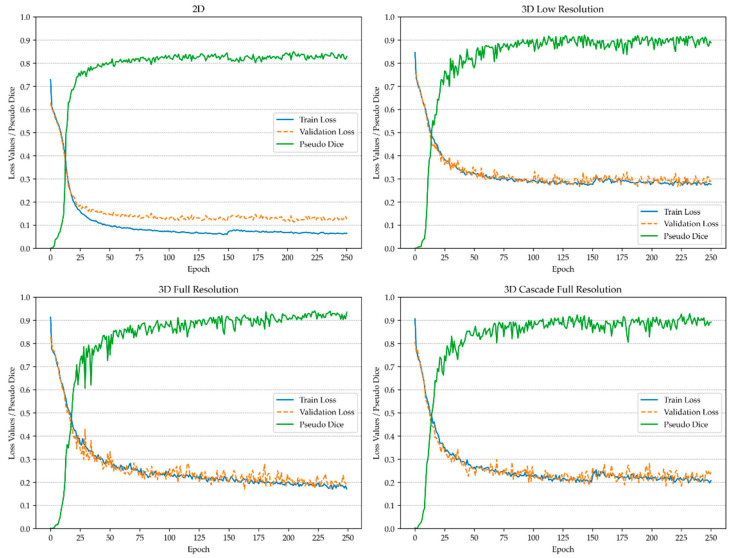
Training process of the model for vertebra detection and instance segmentation.

**Figure 2:**
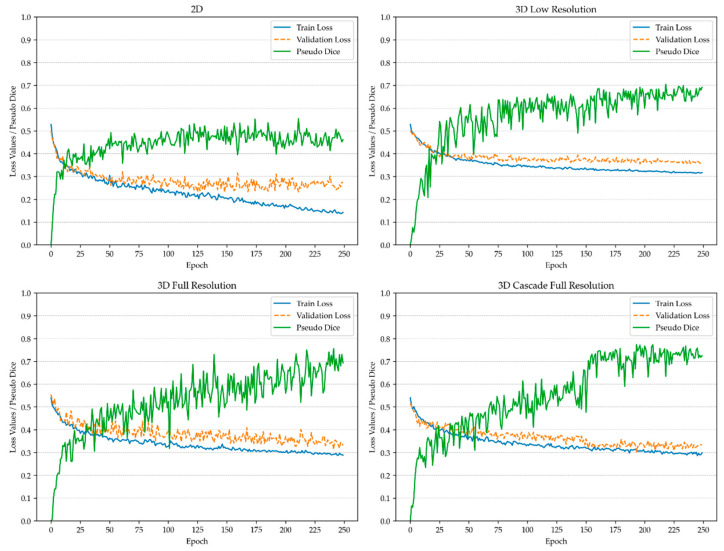
Training process of the model for spinal metastasis detection and instance segmentation.

Validation patches were sampled following the same methodology as during training, enabling a consistent calculation of the Dice coefficient across all sampled validation patches. To monitor the progression of the training and detect potential overfitting, a pseudo-Dice metric was applied, as depicted in Figure 1 and Figure 2. This metric, updated iteratively throughout the training process, served as a preliminary indicator of model performance and differed from the final Dice similarity coefficient, which was computed at the end of training. Unlike the patch-based pseudo-Dice metric, the final Dice similarity coefficient was calculated over the entire image using a sliding window approach, providing a comprehensive assessment of model accuracy on full images.

Performance metrics were derived from validation data using the network architecture that demonstrated the highest performance during training. Metrics related to vertebra segmentation are provided in Table 2, while those for the segmentation of metastatic lesions are detailed in Table 3. This evaluation framework allowed for a robust assessment of model effectiveness in both vertebra and metastasis segmentation, confirming the precision and reliability of the U-Net-based approach for localizing and characterizing metastatic regions within the vertebral column.

## 4. Discussion

Predicting metastasis involves complex spatial and contextual relationships in medical images, which require deep learning models capable of capturing these intricacies. We evaluated several candidate architectures to determine the best performance for our task. VUNet is designed for volumetric (3D) medical data and works well for tasks like organ segmentation in 3D MRI or CT scans [28]. However, our task may involve both 2D and 3D data, and nnU-Net’s versatility in handling both 2D and 3D images with minimal modifications made it more appropriate. SegNet is more lightweight and efficient, making it ideal for real-time segmentation [29]. However, it cannot match the performance of nnU-Net on medical image data, particularly in tasks requiring detailed boundary delineation and high-resolution predictions. nnU-Net’s ability to automatically handle preprocessing and architecture selection gave it a substantial performance edge over SegNet for our medical segmentation task, where accuracy was more critical than speed.

In discussing the model performance, it is essential to address the limitations and suitability of commonly used metrics, such as the Dice similarity coefficient (DSC), particularly in tasks involving small structures like metastases. The DSC, while widely used, may not be an ideal metric for metastasis segmentation, due to its tendency to overemphasize large structures, potentially undervaluing the segmentation accuracy of smaller lesions. Recent literature have suggested that metrics such as F-beta score and panoptic quality offer more reliable performance assessments for instance segmentation tasks [30]. However, the panoptic quality metric, as presented in Table 3, has been criticized for tasks with a high frequency of small, variably shaped segmentations, as it often treats the background as a separate class, complicating its applicability to metastasis segmentation [31].

Given the specific requirements of oncological diagnostics, the F-beta score was customized to better emphasize recall, thereby reducing the risk of false negatives, which are particularly consequential in cancer detection. For this purpose, we adjusted the F-beta score with a beta value of 2, which prioritized recall while maintaining sensitivity to false positives—striking a balance that is critical in detecting metastases, where both false positives and negatives hold clinical significance.

**Figure 3:**
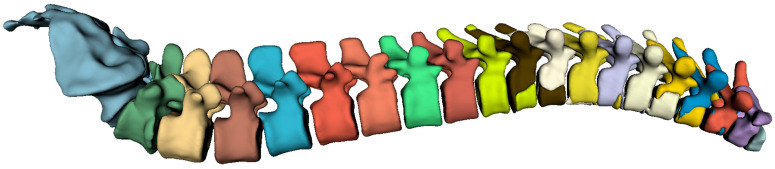
The predicted mask generated by the vertebra segmentation model, with different classes represented in various colors.

**Figure 4:**
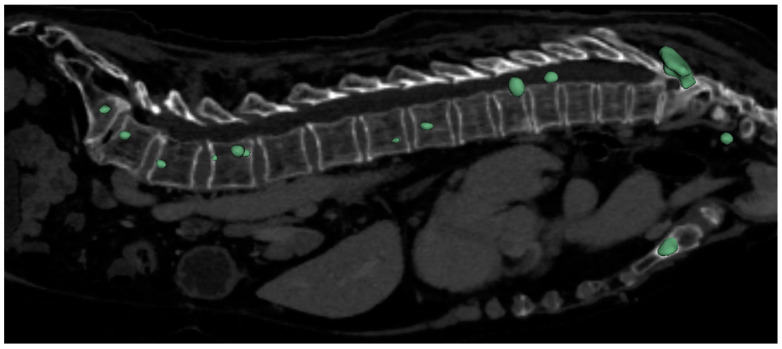
The predicted mask utilizing metastases segmentation model.

Our findings indicate that the 3D full-resolution architecture achieved the highest performance for vertebra segmentation, as illustrated by the predicted mask in Figure 3. Similarly, this architecture demonstrated superior performance for metastasis segmentation, with the results shown in Figure 4. Notably, the model successfully detected and segmented metastases, not only in the spine, but also in other skeletal structures, such as the sternum, as depicted in Figure 4. This capability aligns with recent advancements in deep learning applications for metastasis segmentation, particularly in MRI studies focused on spinal metastases. Our model contributed valuable performance metrics for both lytic and sclerotic metastases, supporting the broader trend of AI-assisted segmentation in oncology.

For lytic metastases, our model achieved a Dice similarity coefficient (DSC) of 0.71, an F-beta score of 0.68, and a panoptic quality of 0.45. These results are comparable to those reported by Kim et al. (2024), who achieved a mean per-lesion sensitivity of 0.746 and a positive predictive value of 0.701 with a U-Net-based model [32]. Additionally, Liu et al. (2021) reported similar segmentation results for pelvic bone metastases, achieving a precision of 0.76 and a recall of 0.67 [33]. Our model’s F-beta score and DSC values indicate a robust capacity for detecting and segmenting lytic lesions, reinforcing the findings from these related studies.

However, the segmentation performance for sclerotic metastases was comparatively lower, with a DSC of 0.61, an F-beta score of 0.57, and a panoptic quality of 0.30. This outcome is consistent with reports in the literature; for example, Ong et al. (2022) achieved a DSC of up to 0.78, with sensitivity rates of 78.9% for sclerotic spinal metastases, highlighting the inherent difficulties posed by the subtle imaging characteristics of sclerotic lesions [22]. Our lower scores for sclerotic metastases reflect these challenges, as the detection and segmentation of sclerotic lesions demand higher sensitivity to subtle density changes, which remains a limitation in current AI models for metastasis detection.

In summary, our research illustrates both the strengths and limitations of a U-Net-based model in detecting and segmenting spinal metastases, providing a balanced assessment across relevant metrics. While lytic lesions were identified and segmented with a high degree of accuracy, sclerotic metastases continued to present challenges. These findings suggest the need for further development of specialized architectures or training strategies tailored to the nuanced imaging characteristics of sclerotic metastases, to improve overall diagnostic accuracy in metastatic spinal disease.

A noteworthy benchmark in vertebrae segmentation is provided by the VerSe: Large Scale Vertebrae Segmentation Challenge, where the state-of-the-art models achieved a mean vertebrae identification rate of 96.6% and a Dice coefficient of 91.7%. This challenge has made a substantial contribution to the field by offering a comprehensive dataset of 374 multi-detector CT scans, catalyzing advancements in vertebrae segmentation research [34]. In comparison, our study utilized a significantly smaller dataset, and the model was trained for a shorter duration (250 epochs). Despite these constraints, our model demonstrated competitive performance, particularly in the segmentation of lytic metastases. However, segmentation of more complex lesion types, such as sclerotic metastases, highlighted areas where further refinement is needed. This comparison underscores the effectiveness of our approach, even within the constraints of limited computational resources and data availability, suggesting its potential for scalability and broader applications.

While our findings are promising, several limitations must be considered. Primarily, the dataset used for training was drawn from a single medical center, which could limit the model’s generalizability across different populations and imaging environments. Expanding the dataset to include a wider array of imaging conditions and patient demographics would likely enhance the model’s robustness and adaptability, contributing to more consistent performance across varied clinical settings. Additionally, the segmentation masks used in training were created manually by medical professionals, a process susceptible to inter-observer variability. This variability could have introduced inconsistencies in the training data, potentially affecting both the model’s learning and the evaluation of its performance. Addressing these issues may involve standardizing segmentation protocols or employing semi-automated segmentation tools to reduce observer-related discrepancies.

Another important limitation is that the model has not yet been validated in real-world clinical environments, where factors such as diverse patient anatomies, varying imaging conditions, and clinical workflow constraints could influence its performance. To ensure the model’s utility and effectiveness in clinical practice, further validation through clinical trials is essential. Such trials would help determine its actual impact on diagnostic accuracy and patient outcomes, providing a more comprehensive assessment of its clinical applicability.

Technical considerations related to CT scanning protocols also play a critical role in model performance. Variability in scanning parameters, particularly resolution, can significantly affect model accuracy. For optimal inference results, the radiological data used should ideally match the technical specifications of the training data, as discrepancies in resolution or imaging quality may reduce model effectiveness. The relatively small dataset for metastasis detection impacted the model’s performance, particularly for sclerotic lesions, underscoring the need for a more extensive dataset to achieve higher reliability. By incorporating a larger, more diverse set of cases with varying patient demographics and imaging characteristics, we could improve both the model’s robustness and its generalizability, ultimately leading to enhanced accuracy in detecting and segmenting metastases across diverse clinical scenarios. This dataset expansion is crucial for increasing the model’s diagnostic effectiveness and realizing its full potential in real-world healthcare applications [35,36].

## 5. Conclusions

This study developed and validated two AI-based models for vertebra and spinal metastasis segmentation, leveraging advanced U-Net architectures to achieve high accuracy across these tasks. Vertebra segmentation demonstrated robust performance, with an F-beta score ranging from 0.88 to 0.96 across vertebra classes, underscoring the model’s precision in anatomical localization. For spinal metastasis detection, the model achieved an F-beta score of 0.68 for lytic metastases and 0.57 for sclerotic metastases, showcasing a strong capacity for identifying lytic lesions, while indicating the greater challenges posed by sclerotic lesion segmentation, due to subtler imaging characteristics.

A significant outcome of this study is the publication of a dataset with annotated CT images containing metastases, which can serve as a valuable resource for future research in metastasis detection and segmentation. This annotated dataset contributes essential information for developing and validating models aimed at metastatic disease, offering a foundational source for further advancements in AI-driven medical imaging.

Notably, the model demonstrated the ability to detect isolated metastatic lesions beyond the spine, such as in the sternum, underscoring its adaptability for broader skeletal metastasis detection and supporting its potential utility across varied clinical scenarios. This capability is crucial for early and accurate metastasis detection, potentially improving patient outcomes by enabling more precise targeting of affected regions.

## Figures and Tables

**Table 1:** Descriptive statistics of the dataset utilized for training the vertebra segmentation model.

Sex	Age	Metastasis Type	Primary Metastatic Site
Female	57	Sclerotic	Melanoma
Female	72	Sclerotic	Lungs
Female	72	Sclerotic	Lungs
Female	68	Lytic	Ovary
Female	39	Sclerotic	Breast
Male	74	Lytic	Prostate
Female	82	Sclerotic	Breast
Female	82	Sclerotic	Breast
Female	64	Sclerotic	Breast
Female	65	Sclerotic	Breast
Female	65	Sclerotic	Breast
Female	61	Sclerotic	Breast
Female	45	Sclerotic	Breast
Female	45	Sclerotic	Breast
Female	70	Sclerotic	Breast
Male	66	Sclerotic	Lungs
Female	52	Sclerotic	Breast
Male	53	Lytic	Kidney
Female	60	Sclerotic	Breast
Male	74	Sclerotic	Blader
Female	79	Lytic	Kidney
Female	48	Lytic	Ovary
Male	66	Sclerotic	Large intestine
Female	73	Lytic	Multiple myeloma
Male	66	Lytic	Multiple myeloma
Female	61	Sclerotic	Breast
Female	73	Lytic	Breast
Female	79	Lytic	Kidney
Female	48	Lytic	Ovary
Male	75	Lytic	Stomach
Male	75	Lytic	Stomach
Male	64	Lytic	Kidney
Female	39	Lytic	Ovary
Male	55	Lytic	Multiple myeloma
Female	60	Lytic	Multiple myeloma
Female	70	Lytic	Breast
Female	32	Lytic	Multiple myeloma
Female	61	Lytic	Kidney

**Table 2:** Metrics for the evaluation of the “3D Full-Resolution” model for vertebra detection and instance segmentation.

Vertebra	Dice Similarity Coefficient	F-Beta Score	Panoptic Quality
C1	0.94	0.94	0.75
C2	0.95	0.95	0.82
C3	0.93	0.93	0.75
C4	0.93	0.93	0.75
C5	0.93	0.94	0.75
C6	0.93	0.93	0.75
C7	0.94	0.93	0.79
T1	0.94	0.94	0.81
T2	0.95	0.95	0.83
T3	0.95	0.95	0.82
T4	0.95	0.95	0.83
T5	0.94	0.94	0.82
T6	0.88	0.87	0.69
T7	0.87	0.88	0.70
T8	0.91	0.92	0.75
T9	0.93	0.93	0.77
T10	0.94	0.94	0.81
T11	0.95	0.95	0.85
T12	0.95	0.94	0.84
L1	0.95	0.94	0.83
L2	0.94	0.94	0.83
L3	0.93	0.92	0.81
L4	0.94	0.89	0.84
L5	0.95	0.94	0.86
Sacrum	0.96	0.96	0.89

**Table 3:** Metrics for the evaluation of the “3D Cascade Full-Resolution” model for metastasis instance segmentation.

Metastasis Type	Dice Similarity Coefficient	F-Beta Score	Panoptic Quality
Lytic	0.71	0.68	0.45
Sclerotic	0.61	0.57	0.30

## Data Availability

CT Scans of Spine with Metastases (Lytic, Sclerotic). https://doi.org/10.5281/zenodo.13645871 (accessed on 25 September 2024).
